# BRD4 inhibition promotes TRAIL-induced apoptosis by suppressing the transcriptional activity of NF-κB in NSCLC

**DOI:** 10.7150/ijms.60776

**Published:** 2021-06-26

**Authors:** Liu Shi, Yu Xiong, Xiaoyan Hu, Zhihao Wang, Conghua Xie

**Affiliations:** 1 Department of Radiation and Medical Oncology, Zhongnan Hospital of Wuhan University, Wuhan, China.; 2 Hubei Key Laboratory of Tumor Biological Behaviors, Zhongnan Hospital of Wuhan University, Wuhan, China.; 3 Hubei Clinical Cancer Study Center, Zhongnan Hospital of Wuhan University, Wuhan, China.

## Abstract

Tumor necrosis factor-related apoptosis inducing ligand (TRAIL) and agonistic antibodies against TRAIL death receptors (DR) can induce apoptosis preferentially in tumor cells while causing virtually no damage to normal cells. However, their therapeutic potential is limited by occurring resistance in tumor cells, including non-small cell lung cancer (NSCLC). Thus, elucidation of the molecular targets and signaling pathways responsible for TRAIL resistance is imperative for devising effective therapeutic strategies for TRAIL resistant cancers. In the present study, we demonstrated that inhibition of Bromodomain-containing protein 4 (BRD4) or genetic knock-down of BRD4, an epigenetic reader and master transcription coactivator, can sensitize lung cancer cells to TRAIL. This sensitization is in a caspase-dependent manner. Inhibition of BRD4 by small molecule inhibitor (+)-JQ-1 and genetic knock-down of BRD4 can both recruit the FADD and activate caspases. The sensitization did not regulate the death receptors DR4 and DR5. Moreover, BRD4 inhibition can block TRAIL-induced IKK activation by suppressing the transcriptional activity of NF-κB. These findings indicate that targeting combination therapy with TRAIL and BRD4 inhibitors can be a promising strategy to overcome TRAIL resistance in NSCLC.

## Introduction

Lung cancer is the leading cause of cancer-related death worldwide and accounts for more than one million deaths per year 1. Lung cancer is assigned to two histological types: small cell lung cancer and non-small cell lung cancer (NSCLC). NSCLC accounts for more than 80% of all lung cancer cases 2. Despite advances in the diagnosis and treatment of NSCLC, the 5-year overall survival rate of NSCLC patients is still extremely low. Therefore, further research is needed to identify new therapeutic targets and tools for the treatment of NSCLC.

In cancer cells, the apoptotic pathway, the cell's natural mechanism for death, is typically inhibited through a wide variety of means. Therefore targeting apoptosis is a promising strategy for anticancer therapy. Tumor necrosis factor-related apoptosis-inducing ligand is a member of the tumor necrosis factor (TNF) family of ligands capable of initiating apoptosis through engagement of its death receptors 3. TRAIL signaling pathway demonstrated a remarkable specificity for inducing apoptosis in tumor cell lines but not in normal cells 4. TRAIL can bind to death receptor-4 (DR4) and death receptor-5 (DR5), the binding of TRAIL with death receptors leads to the trimerization of the death receptors and activation of receptor-mediated death pathway 5. The activated death receptors recruit and activate an adaptor protein called Fas-associated death domain (FADD) through interactions between the death domain (DD) on the death receptors and FADD 5. FADD can recruit and activate caspase-8. In some cell types (type I), activation of caspase-8 is sufficient to trigger apoptosis, whereas, in other cell types (type II), amplification of the extrinsic pathway through the mitochondrial pathway is needed to commit the cells to apoptosis 6. Moreover, TRAIL has been shown to target and induce apoptosis in tumor cells selectively, while sparing normal cells 7. Despite its tremendous potential for cancer therapeutics, the translation of TRAIL into the clinic has been confounded by TRAIL-resistant cancer populations 7. It was found that many cancer cell lines are either intrinsically TRAIL-resistant, or become resistant upon TRAIL treatment. Thus, research on TRAIL resistance is of great importance for ameliorating the therapeutic efficacy and alleviating the suffering of patients.

Bromodomain-containing protein 4 (BRD4) is a transcriptional and epigenetic regulator that plays a pivotal role during embryogenesis and cancer development 8, 9. BRD4 can preferentially localize to “super-enhancer” regions upstream of a variety of oncogenes by interacting with acetylated histones 10. In addition to interacting with acetylated histones, BRD4 has also been shown to promote cancer progression by physically or functionally interacting with transcription factors involved in tumorigenesis, including NF-κB 11, 12. BRD4 maintains constitutively active NF-kB in cancer cells by binding to acetylated RelA 12, 13. BRD4 specific small molecule inhibitor (+)-JQ1 can inhibit the binding of BRD4 to RelA and suppresses NF-kB activation 13. Moreover, several studies showed that TRAIL-mediated apoptosis requires NF-κB inhibition 14, 15. Therefore we speculate that inhibition of BRD4 can overcome TRAIL resistance through regulating NF-κB signaling pathway. Here we showed that inhibition of BRD4 can promote TRAIL-induced apoptosis by suppressing the transcriptional activity of NF-κB in NSCLC cell lines.

## Materials and Methods

### Reagents

Antibodies to BRD4, caspase-8, caspase-3, FADD, DR4, DR5 and GAPDH were purchased from Abcam (Cambridge, MA, USA). Bromodomain Inhibitor (+)-JQ1 was purchased from BioVision (Milpitas, California, USA). rhTRAIL was kindly provided by Shanghai TRAIL Bio-technical Co., Ltd (Shanghai, China). Horseradish peroxidase (HRP)-conjugated goat anti-mouse IgG and goat anti-rabbit IgG were from Southern Biotech (Birmingham, AL, USA).

### Cell culture

Human NSCLC cell lines, including A549 and NCI-H460 (H460), were obtained from the Type Culture Collection of the Chinese Academy of Sciences (Shanghai, China). The cell lines were cultured in RPMI-1640 medium (Hyclone, USA) with 10% fetal bovine serum (FBS), 1% penicillin and streptomycin at 37 °C in an atmosphere of 5% CO2.

### Cell proliferation assay

The effect of (+)-JQ1, rhTRAIL and their combination in A549 and H460 cells was assessed by MTT (3-[4,5-dimethylthiazol-2-yl]-2,5 diphenyl tetrazolium bromide). The MTT assay is used to measure cellular metabolic activity as an indicator of cell viability, proliferation and cytotoxicity. This colorimetric assay is based on the reduction of a yellow tetrazolium salt, MTT, to purple formazan crystals by metabolically active cells. Equal numbers of cells (2000/well) were seeded into 96-well tissue culture plates (Corning, USA). The cells were allowed to adhere overnight before treatment. A blank control group (medium only), a negative control group (untreated cells), and indicated treatment groups were included in this experiment. After incubation at 37 °C for indicated time, MTT dye solution (Beyotime, China) was added to each well. The cell viability was calculated using the following formula: cell viability (%) = (absorbance of the treated wells - absorbance of the blank control wells)/(absorbance of the negative control wells - absorbance of the blank control wells) ×100%. All MTT experiments were performed in five replicates and repeated six times.

### Apoptosis detection

After indicated treatments, cells were harvested by trypsinization and washed with ice-cold PBS (Hyclone, USA) three times. Then, the cells were resuspended in 400 µl binding buffer (Bestbio, China), stained with 5 µl Annexin-V-FTIC (Bestbio) and 10 µl propidium iodide (Bestbio), and incubated at room temperature in the dark for 20 mins. The cells were quantified in a flow cytometer (Beckman). The percentage of apoptotic cells was discriminated from a bivariate histogram of only Annexin-V-FTIC-labeled cells.

### Down-regulation of BRD4 by siRNA

Cells were seeded in six-well plates and transfected with BRD4 siRNA (Gene Pharma, China) using the Lipofectamine 2000 reagent (Invitrogen, USA) according to the manufacturer's instructions. We use 3 individual BRD4 siRNA oligos to knockdown BRD4. BRD4 siRNA (60 pmol)was mixed with 400 µl of serum-free medium and incubated for 5 mins at room temperature; then, 5 µl of Lipofectamine 2000 mixed with 400 µl of SFM was added, and the mixture was incubated for 20 min at room temperature. Finally, the resultant mixture was added to the cells in each well. The medium was changed 6 hours after transfection. The down-regulation of BRD4 by siRNA in cells was confirmed by real-time PCR and Western blot assay 48 hours after transfection.

### Real-time PCR

Total RNA was isolated from cells using TRIzol reagent (Invitrogen). Real-time PCR is carried out using the Power SYBR Green RNA-to-CT 1-Step Kit and 7300 Real-Time PCR System (Applied Biosystems, USA) according to the manufacturer's instructions. The reaction for each sample was performed with 5 replicates.

### Western blotting

For preparation of cell extracts, cells were washed thrice with ice-cold PBS, exposed to the recommended volume of RIPA lysis buffer (Beyotime, China) and placed on ice for 20 min. After centrifugation at 12000 rpm for 5 min at 4 °C, the supernatants were collected and diluted in 5X loading buffer (Beyotime, China). The samples were electrophoresed by SDS-PAGE and transferred to polyvinylidene fluoride membranes. After 2 hours of blocking with 5% fat free milk in TBST (TBS with 0.1% Tween-20) at room temperature, the membrane was incubated overnight with antibodies at 4 °C, washed three times with TBST, incubated for 2 h at room temperature with HRP-conjugated secondary antibodies, and then washed three times with TBST. Finally, immunoreactive protein was visualized with an enhanced chemiluminescence detection kit (Beyotime, China). The levels of target proteins were measured by densitometry.

### Electrophoretic mobility shift assay (EMSA)

Nuclear proteins were prepared by treating cells with lysis buffer (Beyotime, China) according to the manufacturer's protocol. NF-κB-binding activity was assayed using an electrophoretic mobility shift assay (EMSA) kit (Beyotime, China). Double-stranded biotinylated probe was purchased from Beyotime (Shanghai, China). DNA-protein binding was performed at room temperature for 20 mins in a final volume of 20 ml containing binding buffer, double-stranded biotinylated probe and nuclear extract. The DNA-protein complexes were separated by 4% polyacrylamide gel in 0.5×TBE at 100 V at 4 °C for 1 h. DNA-protein complexes in the gel were transferred to nylon membrane (Beyotime) by electroblotting with 0.5×TBE at 100 V for 50 min. DNA-protein complexes were fixed to the membrane by a UV cross-linker and detected using an enhanced chemiluminescence detection kit (Beyotime).

## Results

### The effect of TRAIL on cell proliferation and apoptosis of NSCLC cells

**Figure 1:**
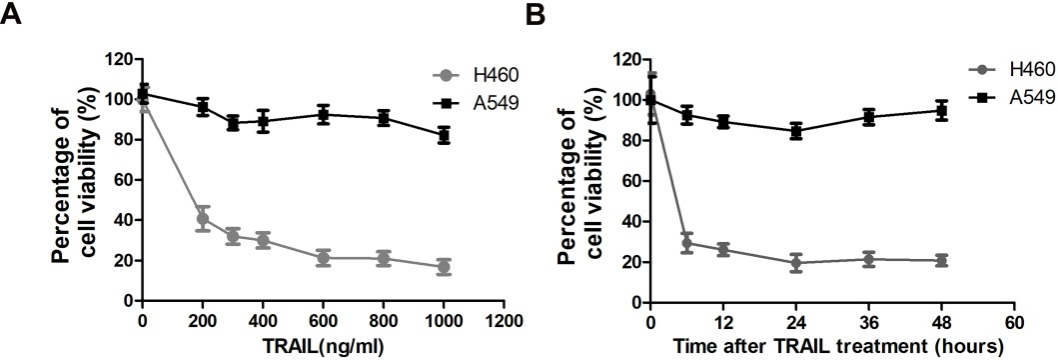
**TRAIL-sensitivity of two NSCLC cell lines. (A)** A549 and H460 cells were treated with indicated concentration of TRAIL for 24 hours. Inhibitory rates were measured by MTT assay. The X-axis represents the TRAIL concentration and the Y-axis represents the percentage of cell viability. **(B)** A549 and H460 cells were treated with 1000 ng/ml TRAIL for indicated time. Inhibitory rates were measured by MTT assay. The X-axis represents the time after TRAIL treatment and the Y-axis represents the percentage of cell viability.

To assess the possibility of TRAIL as a new treatment strategy against NSCLC, we evaluated the sensitivity of two NSCLC cell lines to TRAIL by MTT (3-[4,5-dimethylthiazol-2-yl]-2,5 diphenyl tetrazolium bromide) assay. The MTT assay is used to measure cellular metabolic activity as an indicator of cell viability, proliferation and cytotoxicity. TRAIL treatment leads to reduced viability in NSCLC cell line H460 at very low concentration (200 ng/ml), but has no significant effect on A549 cells even at very high concentration (1000 ng/ml) (Fig. 1A). Thus H460 is a TRAIL sensitive cell line, while A549 is resistant to TRAIL treatment. Furthermore, we also tested the time-dependent response of these two NSCLC cell lines to TRAIL. Fig. 1B shows that the sensitivity to TRAIL in the two NSCLC cell lines exhibited no significant variation after a prolonged exposure time.

### Inhibition of BRD4 sensitizes NSCLC cells to TRAIL

TRAIL is a potential targeted drug for cancer therapy. Moreover, TRAIL is capable of selectively inducing apoptosis of cancer cells. However, the resistance to TRAIL in multiple cancer types resulted in limited response in patients 16. Therefore research on TRAIL resistance is of great importance for ameliorating the therapeutic efficacy. BRD4 has been shown to promote non-small cell lung cancer progression 17. High BRD4 protein levels also have been shown to confer resistance to chemotherapy induced cell apoptosis 18. Here we speculate that BRD4 might be involved in TRAIL resistance.

**Figure 2:**
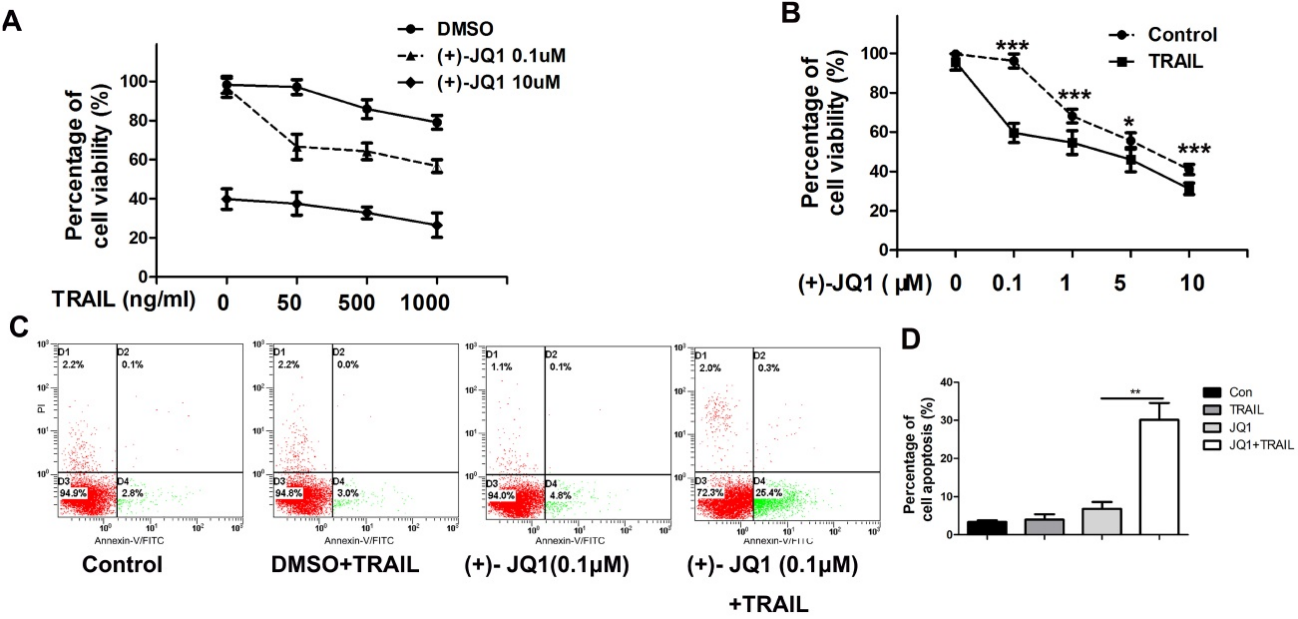
** Effect of (+)-JQ1 on TRAIL induced cell growth inhibition and apoptosis induction in A549 cells. (A)** Cells were treated with either DMSO or the indicated concentration of (+)- JQ1 (0.1 µM and 10 µM) with or without indicated concentrations of TRAIL (from 0 to 1000 ng/ml). Cell viability were measured by MTT assay. **(B)** Cells were treated with either DMSO or the indicated concentration of (+)- JQ1 (0.1 µM and 10 µM) with or without indicated concentrations of TRAIL (from 0 to 1000 ng/ml). Inhibitory rates were measured by MTT assay. **(C and D)** A549 cells were treated with 50 ng/ml TRAIL with or without 0.1 µM (+)-JQ1 for 24 hours, apoptosis were analyzed by flow cytometry after staining with Annexin V-FITC and propidium iodide. Data were analyzed by Student's t-test and shown as mean±SD. *P < 0.05; **P < 0.01; ***P < 0.001.

As shown in Fig. 2A, (+)- JQ1 treatment sensitizes TRAIL resistant A549 cells to TRAIL. Even at very low concentration (0.1 μM), (+)- JQ1 can significantly enhance the killing efficiency of TRAIL in TRAIL-resistant A549 cells (Fig. 2B). Thus, we chose 0.1 μM (+)- JQ1 as a suitable concentration for the remaining experiments. The rate of apoptosis also increased after the co-treatment of these two drugs, as evidenced by increases in the number of Annexin V-positive cells (Fig. 2C). This indicates that inhibition of BRD4 can sensitize NSCLC cells to TRAIL induced cell apoptosis.

### Knockdown of BRD4 by siRNA renders NSCLC cells sensitive to TRAIL-induced apoptosis

**Figure 3:**
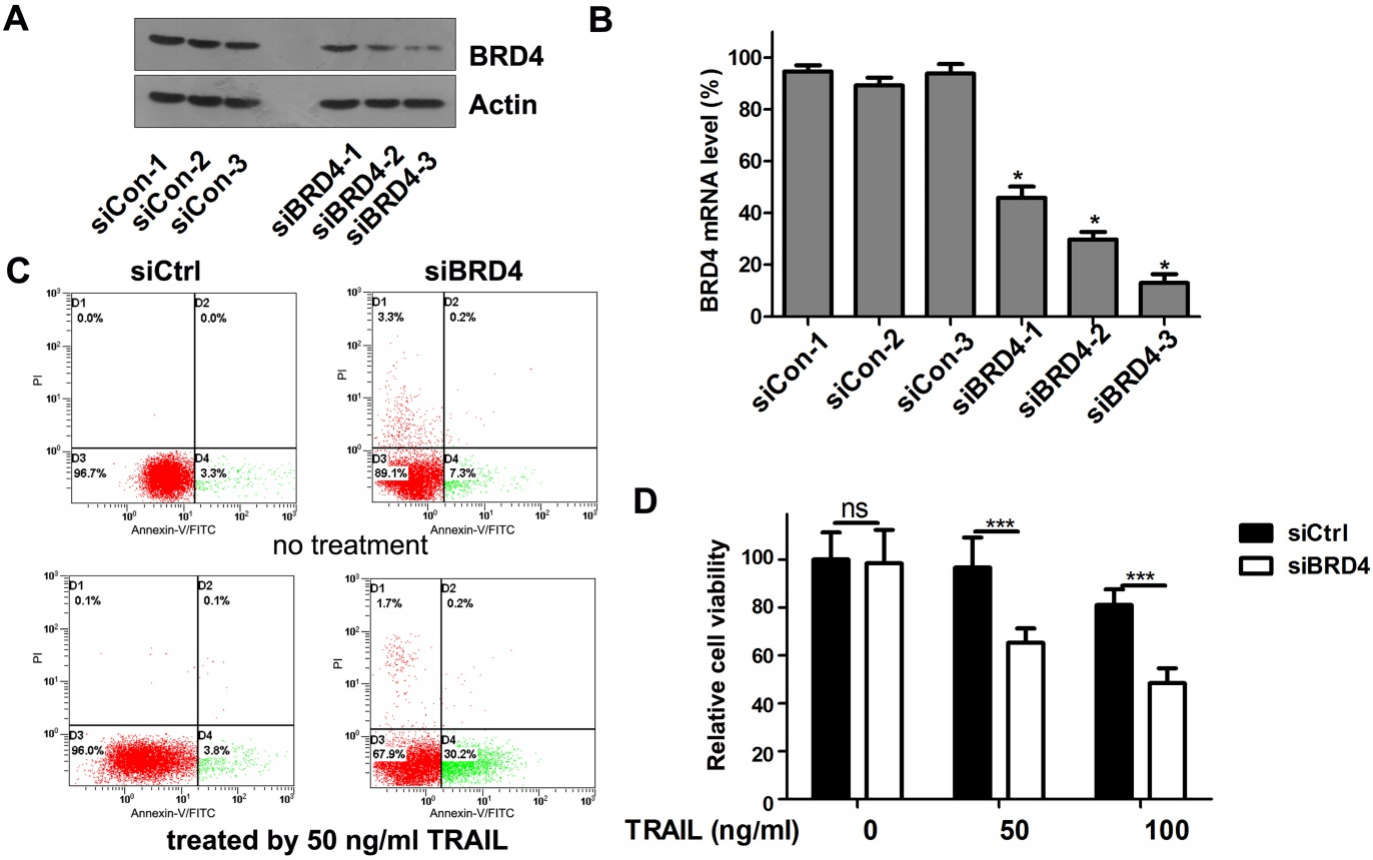
** Effects of BRD4 knockdown on cell growth and apoptosis of A549 cells. (A)** Knockdown efficiency of BRD4 siRNA was detected by western blot. **(B)** Knockdown efficiency of BRD4 siRNA was detected by RT-qPCR., * P<0.05 compared with control. **(C)** A549 cells transfected with BRD4 siRNA-3 were treated with or without 50 ng/ml TRAIL, 24 hours later, cell apoptosis were analyzed by flow cytometry after staining with Annexin V-FITC and propidium iodide for apoptosis. **(D)** A549 cells were transfected with BRD4 siRNA-3 and treated with indicated concentrations of TRAIL (from 0 to 1000 ng/ml) for 24 hours. Cell viability were measured by MTT assay. The difference between controls and cells transfected with BRD4 siRNA-3 was statistically significant. Data were analyzed by Student's t-test and shown as mean±SD. *P < 0.05; ***P < 0.001; ns, not significant.

To test whether the TRAIL-sensitizing effect of (+)- JQ1 was due to BRD4 inhibition, we transfect A549 cells with siRNAs targeting BRD4. The knockdown efficiency was confirmed by western blot (Fig. 3A) and real-time PCR (Fig. 3B). We use 3 individual BRD4 siRNA oligos to knockdown BRD4. All 3 single siRNAs works very well in knocking down BRD4 expression. Among these 3 siRNAs, BRD4-siRNA-3 shows the highest efficiency. So we chose the BRD4-siRNA-3 as the one used for following experiment because it has the highest knockdown efficiency. Cell viability and apoptosis were evaluated. The percentage of apoptosis in TRAIL treated A549 cells was increased to 30.4% in BRD4 knockdown group compared to 3.8% of control group (Fig. 3C). Furthermore, the combination of TRAIL with BRD4 genetic knockdown remarkably reduced the cellular viability compared with the negative control (Fig. 3D). These results support the hypothesis that BRD4 inhibition can sensitize cells to TRAIL induced apoptosis.

### BRD4 inhibition elevates TRAIL-induced FADD expression and caspase-3 activation

**Figure 4:**
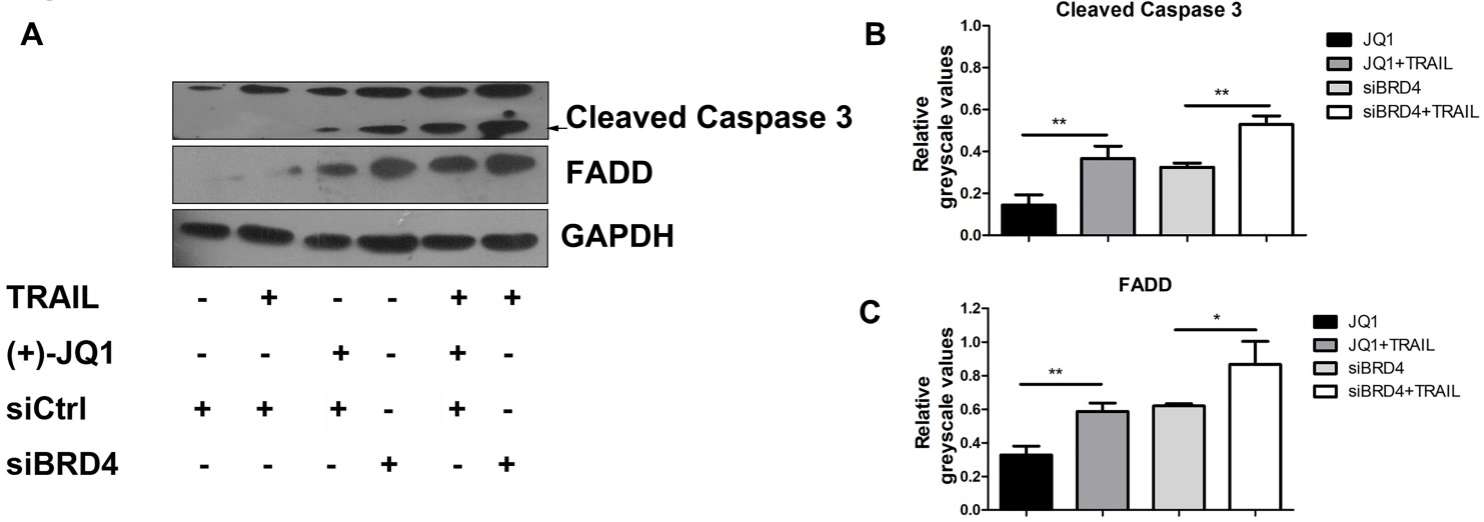
** The effect of BRD4 inhibition or BRD4 knockdown on apoptosis related protein levels. (A)** The protein levels of Caspase-3 and FADD were detected at indicated conditions, black arrow indicates the cleaved Caspase-3. **(B and C)** Greyscale values of bands in (A) were measured by Image J and relative greyscale values to loading control were calculated. Data were analyzed by Student's t-test and shown as mean±SD. *P < 0.05; **P < 0.01.

It is known that TRAIL induces apoptosis via the sequential death signal cascade, which results in the recruitment of FADD to the death domain of receptors and the activation of caspase family proteases 3. Therefore, we next studied the regulatory effect of BRD4 inhibition in TRAIL-induced apoptotic signal events. A549 cells were treated with (+)- JQ1 or transfected with siRNA targeted against BRD4, and were then incubated in culture medium supplemented with or without TRAIL (50 ng/ml). As shown in Fig. 4A, inhibition of BRD4 by (+)- JQ1, or knockdown of BRD4 can increase TRAIL induced induction of FADD and the cleavage of caspase-3 compared with control group. These results show that BRD4 inhibition promotes TRAIL-induced extrinsic apoptotic signal cascades and activates caspases, resulting in a synergistic increase in TRAIL-induced apoptosis.

### Molecular mechanisms of TRAIL sensitization by BRD4 inhibition

**Figure 5:**
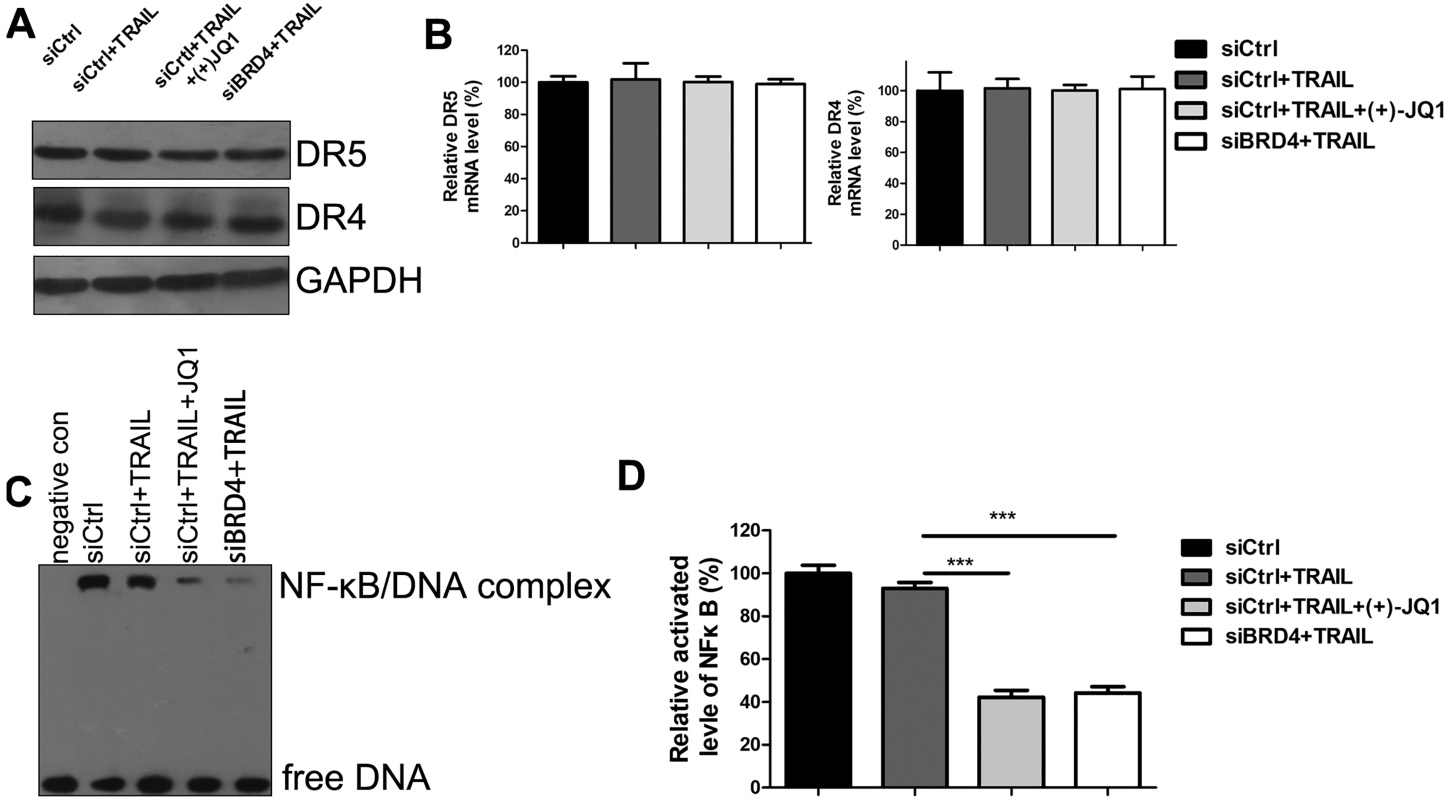
** The effect of BRD4 inhibition or BRD4 knockdown on NF-κB activation. (A)** The protein levels of DR4 and DR5 were detected at indicated conditions. **(B)** The mRNA levels of DR4 and DR5 were detected at indicated conditions. **(C)** The activation of NF-κB were detected by EMSA at indicated conditions. **(D)** Greyscale values of bands in (C) were measured by Image J and relative greyscale values to control were calculated. Data were analyzed by Student's t-test and shown as mean±SD. ***P < 0.001.

To investigate the mechanisms of TRAIL sensitization by BRD4 inhibition, we evaluated the expression of DR4 and DR5 by western blotting (Fig. 5A) and RT-PCR (Fig. 5B). Inhibition of BRD4 by (+)- JQ1 or knockdown of BRD4 doesn't affect DR4 and DR5 mRNA and protein levels. These results indicate that the increased sensitivity to TRAIL by BRD4 inhibition is not due to the expression level change of death receptors. Previous studies showed that TRAIL-mediated apoptosis requires NF-κB inhibition. BRD4 has been shown to maintain constitutively active NF-kB in cancer cells. Furthermore, it has been reported that BRD4 plays an essential role in maintaining constitutively active NF-κB in NSCLC cells. Therefore we speculate that inhibition of BRD4 might sensitize A549 to TRAIL through NF-κB signaling. To test this hypothesis, Electrophoretic Mobility Shift Assay (EMSA) was performed to determine the NF-κB activities. Upon activation, NF-κB can recognize and interact with the unique DNA binding sequence and form DNA/protein complex, which can be separated from free DNA due to the differences in their electrophoretic mobility in non-denaturing polyacrylamide gels. The results showed that NF-κB activities were significantly decreased in the cells treated with TRAIL alone. Moreover, co-treatment with (+)- JQ1 or knockdown of BRD4 caused a further decrease (Fig. 5C/D). These data suggest that the inhibition of BRD4 sensitizes NSCLC cells to TRAIL induced apoptosis by suppressing the activity of NF-κB.

## Discussion

TRAIL induces apoptosis selectively via its interaction with the death receptors DR4 and DR5 in a wide range of cancers, while sparing normal cells 19. Although TRAIL has demonstrated tremendous promise in preclinical studies, the widespread resistance poses significant hurdles for the clinical development of TRAIL-based therapeutics 20. Consequently combined therapy of TRAIL with therapeutic strategies is required to restore the TRAIL sensitivity. Here we showed that inhibition of BRD4 might be a promising strategy to overcome TRAIL resistance in NSCLC. Moreover, we also confirmed that NF-κB pathway is involved in this process.

The selective toxicity of TRAIL against cancer cells makes it an attractive candidate for treating cancers. However, previous studies demonstrated that the majority of NSCLC cells were either partially or completely resistant to TRAIL 21. TRAIL activates apoptosis pathway through directly binding with death receptor (DR4 and DR5), some cancer cells develop TRAIL resistance by expressing low levels of DR4 and DR5 22, 23. In other cancer cells, although the mRNA expression of both DR4 and DR5 is found, there is no correlative link between total receptor expression levels and the sensitivity of tumors to TRAIL 24. There is increasing evidence suggests that activating intracellular anti-apoptosis signal molecules can block the apoptotic signaling pathway 7. Therefore, we focused on exploring other apoptotic signaling mechanisms.

BRD4, member of the Bromodomain and Extraterminal (BET) protein family, is largely acknowledged in cancer for its role in super-enhancers organization and oncogenes expression regulation 25. Inactivating BRD4 or downregulating its expression inhibits cancer development, leading to the current interest in BRD4 as a promising anticancer drug target 26. Previous studies also showed that targeting BRD4 proteins can suppress the growth of NSCLC cells 27, 28. BRD4 inhibitors also have synergistic effects when combined with other chemotherapy drugs 18. Thus we tested whether targeting BRD4 can reverse TRAIL resistance in NSCLC cells. To our surprise, BRD4 inhibition reversed the TRAIL resistance in A549 cells. Previously studies showed that inhibition of NF-κB by blocking activation of the IκB kinase complex reduces Bcl-xL expression and sensitizes tumor cells to TRAIL-induced apoptosis. Brd4 can co-activate transcriptional activation of NF-κB via specific binding to acetylated RelA 12. Thus sensitization of TRAIL resistant NSCLC cells by BRD4 inhibition is at least partially through NF-κB pathway. Here we also found that BRD4 inhibition doesn't regulate the mRNA and protein expression levels of DR4 and DR5. Thus, these results indicate that BRD4 inhibition mediated TRAIL sensitization is not directly linked to the regulation of DR4 and DR5.

Previous literature has reported that TRAIL can bind to DR4 and DR5, leading to the recruitment of adaptor proteins to form the death-inducing signaling complex (DISC) that contains Fas-associated death domain (FADD) and the apoptosis initiating protease pro-caspase-8 (or pro-caspase-10) 7. Within the DISC, pro-caspase-8 or pro-caspase-10 is auto-catalytically cleaved and releases active caspase-8 or caspase-10 into the cytoplasm, which leads to the cleavage and activation of effector caspases, such as caspase-3, caspase-6 and caspase-7, in the extrinsic apoptotic pathway 4. To determine the regulatory effect of BRD4 inhibition in TRAIL-induced apoptotic signal events, we tested the FADD and cleaved fragment of caspase-3 and caspase-8. We found that BRD4 inhibition elevates the TRAIL-induced FADD expression and caspase-3 activation.

In summary, we found that targeting BRD4 can sensitize NSCLC cells to TRAIL induced cell proliferation inhibition and apoptosis induction, which occur partly through the inactivation of the NF-κB pathway. Therefore, inhibition of BRD4 might be a potential strategy for treating NSCLC and overcoming resistance to TRAIL.
